# Bacterial contamination of drinking water sources in rural villages of Mohale Basin, Lesotho: exposures through neighbourhood sanitation and hygiene practices

**DOI:** 10.1186/s12199-019-0790-z

**Published:** 2019-05-15

**Authors:** Patrick Gwimbi, Maeti George, Motena Ramphalile

**Affiliations:** Department of Environmental Health, National University of Lesotho, Roma, 180 Lesotho

**Keywords:** Drinking water, Neighbourhood sanitation, *E. coli*, Hygiene practices, Mohale Basin, Lesotho

## Abstract

**Background:**

Bacterial contamination of drinking water is a major public health problem in rural areas of sub-Saharan Africa. Unimproved water sources are a major reservoir of *Escherichia coli* (*E. coli*) causing severe diarrhoea in humans. This study assessed *E. coli* counts in drinking water from different sources and their relationship with water source protection status and neighbourhood sanitation and hygiene practices in rural villages of Mohale Basin in Lesotho.

**Methods:**

Thirty drinking water sources were purposively sampled and their water analysed for *E. coli* counts. The types of water sources, their protection status and neighbourhood sanitation and hygiene practices in their proximity were also assessed. *E. coli* counts in water samples were compared to water source protection status, neighbourhood sanitation, hygiene practices, livestock faeces and latrine proximity to water sources.

**Results:**

*E. coli* counts were found in all water samples and ranged from less than 30 colony-forming units (cfu)/100 ml to 4800 cfu/100 ml in protected sources to 43,500,000 cfu/100 ml in unprotected sources. A significant association between *E. coli* counts in drinking water samples and lack of water source protection, high prevalence of open defecation (59%, *n* = 100), unhygienic practices, livestock faeces and latrine detections in proximity to water sources was found in the study (*P* < 0.05).

**Conclusion:**

Water sources in studied villages were contaminated with faeces and posed a health risk to consumers of that water. Community-led sanitation and hygiene education and better water source protection are urgently needed.

## Introduction

Bacterial contamination of drinking water is a major contributor to water-borne diseases in rural areas of most developing countries where water sources are communally shared [1, 2] and exposed to multiple faecal-oral transmission pathways in their neighbourhood boundaries [3, 4]. *Escherichia coli* (*E. coli*) infections associated with drinking contaminated water remain a major public health concern as its presence signifies fatal illnesses such as diarrhoea [3, 5]. The World Health Organization (WHO) estimates that diarrhoeal disease due to exposure to unsafe drinking water, inadequate sanitation and hygiene practices contribute to more than 25% of reported global environmental burden of the disease [6]. Given this status, highly effective interventions for prevention and control of *E. coli* contamination of water sources are essential.

In sub-Saharan Africa, with deteriorating environments attributed to high levels of open defecation, drinking water sources remain vulnerable to faecal contamination [6, 7]. Approximately 215 million people practice open defecation [7], a major source of transmission mode of pathogens that cause diarrhoeal diseases. *E. coli* is the most common cause of diarrhoeal diseases infections as well as human gastrointestinal tract infections caused by ingestion of unsafe drinking water in children in low-income countries [8]. According to Gizaw et al. [8], a greater proportion of intestinal parasitic infections in sub-Saharan African countries is associated with poor water, sanitation and hygiene conditions and most of the infections are faecal-oral. Harris et al. [6] also characterised the relationship between sanitation and the risk of water contamination in rural Mali, concluding that *E. coli* concentrations in communal water sources were significantly associated with poor neighbourhood sanitation practices. Poor socio-economic status of communities enhances and/or increases open defecation rates and unhygienic practices increasing the transmission of bacterial pathogens into water sources [9]. Accordingly, there is a need to explore neighbourhood sanitation and hygiene practice and water quality at source, as well as the exposure-response relationship.

In Lesotho’s rural areas, diarrhoea illnesses are a severe public health problem and a major cause of morbidity and mortality in infants and young children and most of it is related to faecal pollution of water sources from poor environmental hygiene and sanitation [10]. Approximately 70% of the population practice open defecation, and it is estimated that the child mortality rate was 85.9 deaths per 1000 live births in 2017 [11, 12]. Exposures to faecal contamination occur at the community-scale via contaminated public water sources originating from within the community. However, transmission pathways of pathogens into the water sources, other than household sanitation, remain understudied even though these could present major sources of faecal exposure. Knowledge of such exposures to faecal contamination of water sources has implications for the number of potential source(s) of pathogen exposure and how interventions may need to be designed, delivered and measured.

The objective of this study was to evaluate *E. coli* counts in drinking water from selected communal water sources and their relationship with water source protection status and neighbourhood sanitation and hygiene practices in rural villages of Mohale Basin in Lesotho.

## Materials and methods

### Study area

**Fig. 1:**
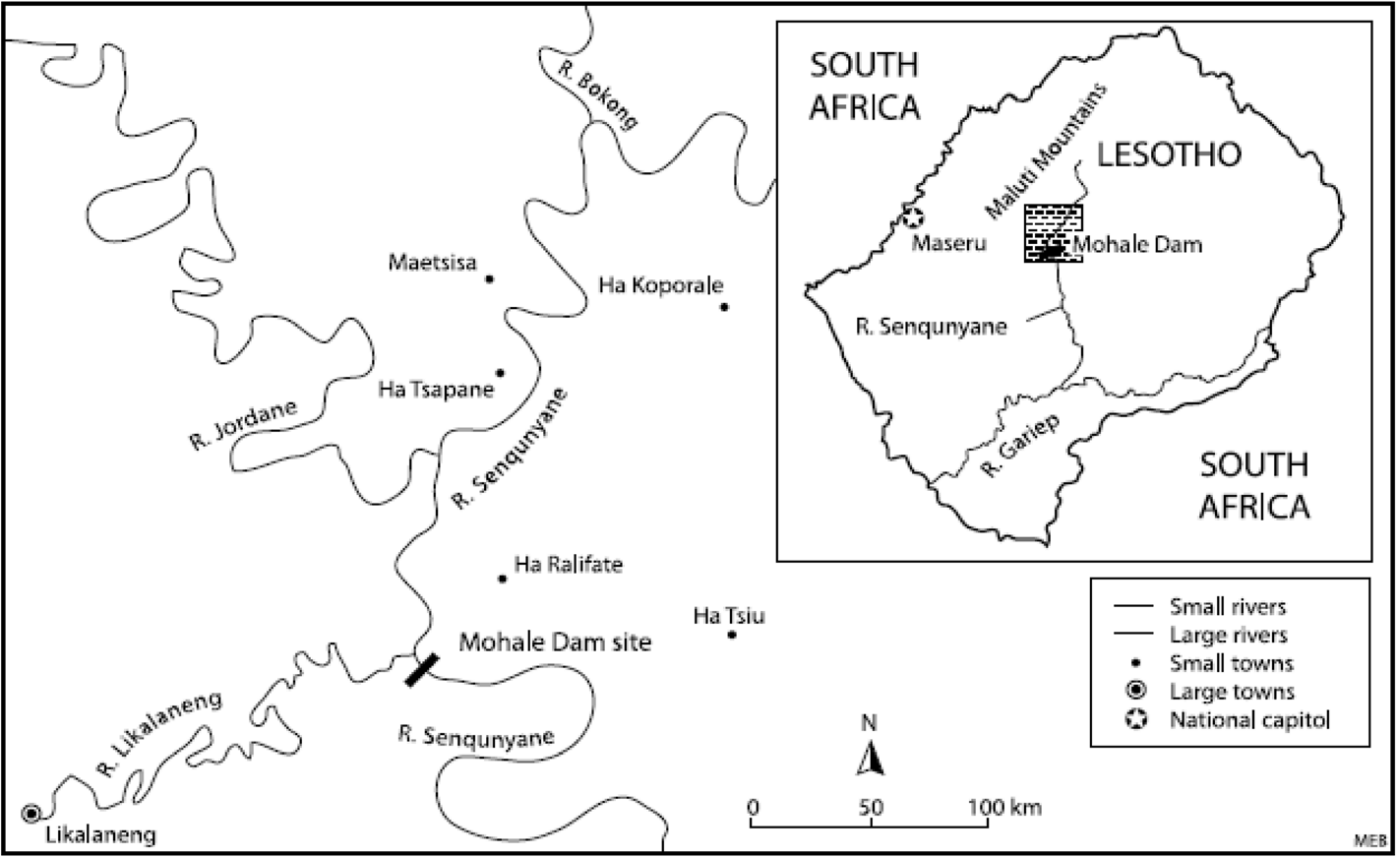
Mohale Basin. Source: [9]

Figure 1 shows the general location of the study area. Mohale Basin is the term applied to the area affected by the construction of Mohale Dam in the Lesotho Highlands [13] under the Lesotho Highlands Water Project (LHWP). The LHWP is a multi-phase water transfer and hydro-electric power scheme that diverts water from Lesotho’s Senqu River system to the upper reaches of the Vaal River in South Africa [14]. The study area is characterised by an elevation of between 2500 and 3000 m above sea level. The general landscape is that of high to very high relief with some grassland, shrub land, wetlands, cultivation and settlements [14].

The context of communities investigated in the study is that of people who manage their lives through subsistence farming under rural-life settings. The communities live along the valleys of the Senqunyane, Jordane and Bokong Rivers (Fig. 1) in Thaba-Tseka district. Following the construction of the Mohale Dam, some 1900 people from 14 villages with 321 households were resettled in different places. Some however opted to remain in the vicinity and relocated to up-slope areas [13]. Livestock ownership is commonly an indicator of a household’s economic status, and there is a wider distribution of cattle among households. The livestock and homesteads are generally located upstream of the different water sources. A common feature of the community is summer grazing where livestock owners use cattle post areas in the mountains during the summer months [14]. It is commonly the residents of the adjacent villages who have preferential access [15].

The main sources of drinking water are natural springs, though 44% of the villagers in the previous study indicated that they used river water regularly for drinking purposes (Reilly, 2009). The average distance from water sources to households varies from 200 m to 7 km from sources. The water sources are potential habitats for microbial pathogens due to high prevalence of open defecation and livestock faeces contamination [10]. Previous studies have revealed that water from the Mohale dam and inflows from its tributaries is not as free from contaminants as had been thought [16]. Common water-borne ailments include diarrhoea and stomach ache [14, 16].

Ventilated improved pit (VIP) latrines to improve households’ sanitation were introduced in the communities with assistance from the Lesotho Highlands Development Authority [14]. the average distance from latrines to protected water sources sampled was 15 m. Eligible communities received water, sanitation and education interventions from the Lesotho Highlands Development Authority after the construction of Mohale Dam.

### Research design

This study was cross-sectional in design. Data collection used mixed methods. The research population consisted of drinking water from different water sources used by households in the Mohale Basin and households under the jurisdiction of three chiefs in the same area. The relationship between *E. coli* counts in drinking water and type of water sources used by households and views of households on neighbourhood open defecation, hygiene practices, livestock faeces and latrine detections in proximity to water sources were analysed.

Water samples were purposively collected from 30 different water sources. Water sources were first put into categories based on their level of protection and type of source, namely: protected springs, unprotected springs, protected open wells, unprotected open wells, streams and others representing boreholes and rainwater during the household survey. Thirty water sources were then purposefully and proportionally selected to represent the geographical distribution of the study area and the different categories of drinking water sources used by households. Due to budget constraints, it was not possible to evaluate all the water sources used by all the households surveyed. Water samples were collected in May 2018 and examined for *E. coli* counts.

Households’ views on their neighbourhood defecation and hygiene practices were evaluated through questionnaires. Households to be interviewed were identified using a multi-stage cluster sampling technique. In the first stage, the list of all villages in the study area was established with the help of local community leaders. In the second stage, the number of households in each village to be surveyed was determined using the probability proportional size. In the final stage, the household to be interviewed in each village was selected using systematic random sampling. One hundred households under the jurisdiction of three chiefs in Mohale Basin were selected to represent the area under the three chiefs. Sample participants were characterised as adults from 18 years and above, who resided in the study areas and could understand and answer questions that were asked.

### Data collection

Each water sample was collected in a sterile 100-ml bottle and subsequently tested for *E. coli* counts within 24 h of collection. To ensure sample preservation, the bottles with water samples were placed in a cooler box with ice packs immediately after collection in order to maintain 4 °C and then transported to the laboratory for analysis within 24 h owing to the long distance between sampling points and the laboratory.

Questionnaires were administered to residents of the study area who used water sources in order to obtain information on neighbourhood defecation and hygiene practices and to assess their knowledge of the safety of their water for drinking.

### Determination of *E. coli* counts in water samples

The standard plate count method was used to determine the *E. coli* counts. The methodology for the Petrifilm plates was used, but samples were passed through sterile 0.45-μm filters prior to incubation. Sample incubation involved inoculating and spreading 1 ml of water sample on the gel, then incubating the plates at of 35 °C for 24 h, and counting the number of blue colonies associated with a small gas bubble. Cultures were enumerated by counting the number of blue colonies. This test was also used to determine the most probable number of *E. coli* per 100 ml of water.

### Data analysis

Data were analysed using STATA 14 software. Data from the structured questionnaires were descriptively analysed and presented using graphs and tables. To assess the degree of bacterial contamination by type of water source, *E. coli* counts found from different water sources were compared.

Multivariable logistic regression analyses were performed to examine factors associated with faecal contamination of water samples at source, including, type of source water protection, neighbourhood open defecation and hygienic practices, livestock faeces and latrine citations in proximity to water sources. Pearson correlation was used to establish whether there was a statistically significant relationship between *E. coli* counts in water and water source protection status, neighbourhood open defecation and hygienic practices and livestock faeces at 5% level of significance.

### Ethical approval

Ethical clearance was obtained from the National University of Lesotho Ethical Review Board of the Faculty of Health Sciences. Informed consent was sought and obtained from each respondent before data collection commenced. Confidentiality was assured by not using names and keeping questionnaires anonymous.

## Results

### Households’ perceptions of water safety at source

All households collected their drinking water from nearby communal water sources (Table 1). The proportion of households drinking from unprotected springs was the highest (67%), followed by those using protected springs (24%), then open wells (6%) and lastly streams (3%).

**Table 1:** Types of water sources used by households

Type of water source	Frequency	Percentage
Unprotected springs	67	67
Protected spring	24	24
Open well	6	6
Stream	3	3

Overall, 77% of the households were satisfied that their water was safe for drinking without treatment, while 23% indicated that it was not safe. Perceived risks of water contamination depended on the source of water used by the household. The highest perception of water being safe was recorded among the protected springs users (81%, *n* = 24), followed by those using unprotected springs (67%, *n* = 67), then open wells (63%, *n* = 6) and lastly streams (33%, *n* = 3).

Most residents understood the quality of water in terms of clarity, colour, smell and composition of the water.

Diarrhoeal disease was named the leading cause of illness by a majority of the households (70%) and was said to occur mostly in the rainy season suggesting poor water source protection as a potential mechanism for source contamination. Only one respondent mentioned typhoid as the other diseases resulting from poor water quality.

**Fig. 2:**
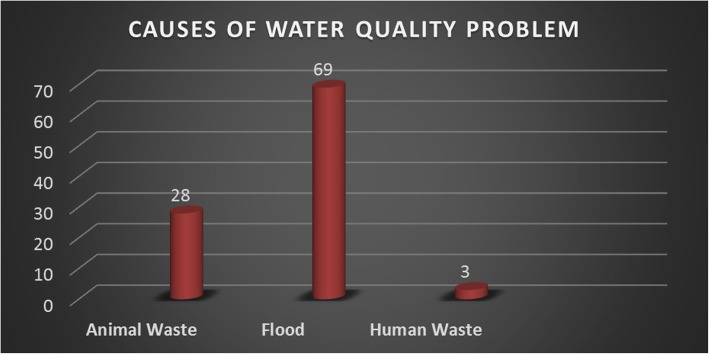
Factors perceived to be the key causes of water contamination at household level

Households’ views on the causes of water contamination are shown in Fig. 2. Flooding was the dominant pathway of exposure to faecal contamination regardless of source water protection (69%), followed by animal waste (28%) and lastly open defecation (3%).

Other water-related challenges experienced by households included drying up of sources during the dry season (98%) and lack resources to properly protect water sources from animals (28%).

### Prevalence of *E. coli* in water samples at source

The water sources consisted of 13 unprotected springs, 9 protected springs, 4 unprotected open wells, 2 protected open wells and 2 stream water sources samples (Table 2). The majority of water sources were located down slope of high ground, with settlements and latrine facilities at elevated ground raising concern that leaching of excreta from latrines could be adversely impacting underlying groundwater resources upon which households are dependent on for their domestic water supply.

**Table 2:** Occurrence of *Escherichia coli* in drinking water for humans

Source of water	Total number of samples tested	Range of *Escherichia coli* counts per 100 ml	Mean *Escherichia coli* count per 100 ml
Protected spring	9	< 30–4.2 × 10^3^	493.3
Unprotected spring	13	4.5 × 10^3^–4.35 × 10^7^	13,017.7
Protected open wells	2	< 30–5 × 10^3^	2515
Unprotected open wells	4	5.5 × 10^3^–9.8 × 10^3^	7650
Stream	2	1.15 × 10^4^–1.18 × 10^4^	11,650

All water samples were positive for *E. coli* contamination. The *E. coli* counts ranged between < 30 and 4.35 × 10^7^ cfu/100 ml. Water source protection status, location with respect to latrine, proximity to open defecation and unhygienic practices were significant determinants of variability in *E. coli* counts in different samples. *E. coli* counts of 4.2 × 10^3^ cfu/100 ml were detected in one such spring despite being categorised as protected.

The concentrations of *E. coli* in water samples from unprotected water sources were significantly higher than those from protected ones (*p* < 0.05). Unprotected open wells and streams showed the highest contamination with average *E. coli* counts in excess of 11,650 cfu/100 ml (Table 2). A greater proportion (78%) of samples from unprotected water sources had *E. coli* counts exceeding 4.3 × 10^7^ cfu/100 ml. In comparison, *E. coli* counts in 60% of the samples from protected sources (*n* = 9) had less than 30 cfu/100 ml.

Higher values of *E. coli* counts were observed in samples from open wells and streams compared to unprotected springs. The protected springs had a spring box with an overflow pipe but with no fence around the spring. Animals could access the spring. There was no diversion ditch above the spring.

WHO recommends the health risk categories of *E. coli* counts in drinking water to be 0 cfu/100 ml (conformity), 1–10 cfu/100 ml (low risk), 10–100 cfu/100 ml (intermediate risk), 100–1000 cfu/100 ml(high risk), and > 1000 cfu/100 ml (very high risk) [17]. These results indicate that for the households of Mohale studied, the probability of having a human health concern due to consumption of this water is widespread especially as indicated by *E. coli* counts. The *E. coli* counts could indicate a greater risk of water consumers developing diarrhoea disease infections through drinking the untreated water.

### Neighbourhood sanitation and hygiene practices in proximity to water sources

Fifty-nine percent of the respondents stated that they regularly practiced open defecation (Table 3). While the majority indicated that they owned latrines (62%, *n* = 100), only 41% indicated that they regularly used them over 24 h. The other 21% stated that latrine use did not suite their daily routine activities as they spent most of their time away from home.

**Table 3:** Sanitation practices among households

Latrine use by households	Frequency	Percentage
All the time	24	24
Most of the time	17	17
Sometimes	21	21
Never	38	38

Lack of laundry facilities in proximity to water sources had an effect on water contamination as 78% (*n* = 100) of the respondents indicated that they washed their clothes and bathed along rivers or near water collection points. Visual inspections showed that generally such water sources were located on downstream of the activity points.

More than 80% of the respondents reported that they washed their containers before fetching water. The majority (55%) of them used water and soil to wash their containers (Table 4). Households dipping their containers in open wells during water collection could be significantly increasing *E. coli* counts in water sources as some containers were not clean.

**Table 4:** Materials used to clean storage containers by household before water collection from sources

Material used to clean storage container before water collection from source	Frequency	Percentage
Soap and water	27	27
Sand and water	55	55
Water Only	18	18
Total	100	100

The common source of faecal contamination in drinking water sources appears to be open defecation, livestock faeces, infiltration of faecally contaminated water from nearby latrines, inadequate protection of water sources and unhygienic management of sources.

## Discussion

This study demonstrated that the quality of water at source was compromised by poor neighbourhood sanitation and unhygienic practices. *E. coli* counts were detected in all drinking water samples and were significantly related to high prevalence of open defecation (59%, *n* = 100) and unhygienic practices in proximity to water sources, lack of source water protection and source proximity to pit latrines. These results concur with findings reported in Ethiopia [8, 18], northern Pakistan [17], Zimbabwe [19] and the Shashemane Rural District of Ethiopia [20] which all pointed to high counts of *E. coli* in drinking water sources due to poor neighbourhood sanitation and hygiene practices around water sources and failure to protect water sources. Studies in Zimbabwe attributed *E. coli* counts in water sources to livestock faeces entering drinking water sources [19], while in the Shashemane District in Ethiopia, open defecation [20] was the main contributory factor. Gizaw et al. [8] similarly noted that 70.2% of the households in rural Dembiya in northwest Ethiopia consumed water which was not good for humans and households were at a high risk of being infected with intestinal parasites caused by helminths and protozoa. The high prevalence of *E. coli* in water samples reported by this cross-sectional study may be due to the fact that the area is characterised by poor sanitation and hygiene conditions. A greater proportion of households practiced poor sanitation and hand washing, and most households accessed water sources exposed to such poor practices.

This study also revealed that high runoff increased the risk of diarrhoea among households (70%, *n* = 100), suggesting exposure of water sources to runoff-washed infections. Previous literature has suggested associations between rainfall patterns and diarrhoea [21, 22]. A study in Ibadan, Nigeria, for example reported an association between high rainfall pattern and an increased risk of diarrhoea [21]. Mukabutera et al. [22] also reported a significant association between presence of diarrhoea among children under 5 years of age in households and rainfall pattern in Rwanda. The outcome of this study about the association between *E. coli* counts in water samples supports these results. The impact of rainfall patterns on diarrhea is likely to be most extreme when sanitation is compromised. Kravitz et al. [23] previously found that less than 5.0% surveyed villagers in the study area used latrines and as a result, the prevalence of water-borne diseases especially diarrhoeal diseases was very high. In future studies, comparing *E. coli* counts among different types of both unimproved and improved water sources should be an important consideration.

The relatively low *E. coli* counts in water samples from protected sources demonstrate the importance of adequate source water protection in safeguarding water quality. The risk of source water contamination is a human health concern in the study area given the study findings. Ibrahim and Patrick [24] suggest that drinking water contamination can be avoided through greater attention to land use practices aimed at the protection of public water supplies. Source water protection is recognised as the first barrier in the multi-barrier approach to reduce the risk of drinking water contamination [24]. The critical role of governments in supporting source water protection planning and management through policies legislation and strategies and in rural communities needs to be reviewed in line with the study findings. The current lack of human capacity at community level to support source water protection implementation suggests the need for the government to help local communities build the capacity to protect their water sources.

The results also underscore the importance of monitoring source water in addition to putting emphasis on community-led sanitation when implementing water, sanitation and hygiene interventions in rural communities. The *E. coli* counts exceeded the WHO public health risk categories of 0 cfu/100 ml(conformity), 1–10 cfu/100 ml (low risk), 10–100 cfu/100 ml (intermediate risk), 100–1000 cfu/100 ml (high risk), and > 1000 cfu/100 ml (very high risk) [17]. Previous studies have shown that when communities are informed about risks to their water, they adjust their practices to ensure its safety [25]. However, more research is needed on the content and delivery of such information to affect sustained behaviour change.

The detection of *E. coli* counts in all drinking water samples indicates significant widespread faecal contamination of water sources in the neighbourhood domain of water sources. They point to the need for research and interventions focused on reducing their exposures to human and livestock faecal pathogens as well as personal and domestic hygiene in the study area. Developing standards for latrine siting in terrains such as Lesotho would provide a welcome addition in rural areas where this is a major challenge. Public latrines should also be promoted near public water sources.

Other options such as point-of-collection chlorination have shown promise in improving water quality [25]. More studies are needed to identify appropriate and effective solutions with scalable promise, while concurrent efforts are underway [25].

## Conclusion

*E. coli* contamination of water sources are an important factor contributing to the high incidence of diarrhoea in Lesotho’s rural areas. This study showed that *E. coli* counts in water samples from different sources were associated with source water protection status and poor neighbourhood sanitation and hygiene condition. All 30 water samples obtained from 30 different water sources had *E. coli* counts ranging from < 30 to 4.35 × 10^7^ cfu/100 ml. Sources of faecal contamination were largely inadequate source protection, high prevalence of open defecation, livestock faeces exposure, unhygienic practices and latrine proximity to sources of water. Because of the high *E. coli* counts in water, regular water quality monitoring, combined with community-led intervention with a focus on sanitation, hygiene education, better source water protection strategies and source water treatment, is recommended. Future studies should sample more water sources throughout the Mohale Basin as this study was confined to few sampled sites to determine the degree of bacterial contamination of water sources.

## Abbreviations

cfu: Colony-forming units
*E. coli*: *Escherichia coli*
LHWP: Lesotho Highlands Water Project
VIP: Ventilated improved pit
WHO: World Health Organization
